# Fast preparation of RG-I enriched ultra-low molecular weight pectin by an ultrasound accelerated Fenton process

**DOI:** 10.1038/s41598-017-00572-3

**Published:** 2017-04-03

**Authors:** Zijian Zhi, Jianle Chen, Shan Li, Wenjun Wang, Rui Huang, Donghong Liu, Tian Ding, Robert John Linhardt, Shiguo Chen, Xingqian Ye

**Affiliations:** 1 Zhejiang Key Laboratory for Agro-Food Processing, Department of Food Science and Nutrition, Fuli Institute of Food Science, Zhejiang University, Hangzhou, 310058 China; 2 Center for Biotechnology & Interdisciplinary Studies, Department of Chemistry & Chemical Biology, Rensselaer Polytechnic Institute, Biotechnology Center 4005, Troy, NY 12180 USA

## Abstract

Pectin, a natural polysaccharide found in the cell wall of most higher plant such as citrus, has drawn much attention due to its potential beneficial role in facilitating the treatment of many diseases like cancer, hyper cholesterol and diabetes. However, the broad application of pectin faces great limitations as the large molecular size of pectin severely prevents its bioavailability *in vivo*. In this study, we report an effective and highly convenient approach to degrade natural pectin into lower molecular pectin. By combining ultrasound with Fenton system (US-Fenton), we show that ultrasound synergistically enhances the efficiency of Fenton reaction to degrade pectin into 5.5 kDa within only 35 minutes. Importantly, RG-I domain, the most effective portion of natural pectin, was well preserved and highly enriched. In addition, the antioxidant activities of US-Fenton-treated pectin was significantly elevated. The mechanism of this novel observation was further investigated through the multiple structural analyses including HPLC, IR and NMR. Taken together, we present a novel and convenient approach to generate ultra-low molecular weight pectin with high efficiency and higher bioactivity. We expect our approach will have broader applications in improving the bioavailability and bioactivity of other polysaccharide-based natural compounds.

## Introduction

The name of pectin was originated from Ancient Greek, which means “congealed or curdled”. Pectin is a form of polysaccharide with the most complicated structure found so far in the middle lamellae and primary cell walls of higher plants. Under normal physiological condition, pectin function as a hydrating agent and cementing material to support the cellulosic network^1^. In the food and other industry, pectin has been widely used as a texturizer, stabilizer, and emulsifier^1–3^. Recently much more attention has been drawn on this interesting natural compound as its potential applications in healthcare to reduce the risk of cancer^4^, hypercholesterolemia, diabetes, obesity and to maintain intestinal health^5^.

Nature pectin is a highly heterogeneous form of polysaccharide with huge molecular size. It contains multiple “domains or building blocks” varying from linear-form homogalacturonans (HG) to highly branched-form rhamnogalacturonan-I (RG-I) and rhamnogalacturonan-II (RG-II). RG-I and RG-II can be distinguished based on their branching degrees and the composition of side-chain monosaccharides within them^6^. It has been reported that the *Lonicera japonica* flower-derived RG-I -rich pectin can significantly inhibit the *in vitro* growth of several pancreatic cancer cell lines^7^. Therefore, RG-I domain has been considered as the most important region in pectin because of its enrichment of pectin’s immune-modulating and anti-tumor bioactivities.

Despite the evident beneficial effect of pectin RG-I domain *in vitro*, the intact natural pectin exhibits marginal bioavailability and limited bioactivity^8^ *in vivo*. One possible reason to explain its discrepancy is that the large size of natural pectin prevents its RG-I content from being directly absorbed by the body. This assumption was supported by the finding that acid-treated pectin exhibited significantly higher inhibitory effect on the metastatic of a spectrum of cancer cell line than the nature pectin, most likely due to the degradation into small fragments^9^. Therefore, to increase its *in vivo* bioavailability, low molecular weight pectin (LMP) needs to be prepared to release to its RG-I region.

Chemical^10–12^ and enzymatic modifications^13, 14^ as well as physical treatment such as heat^11, 15^, ultrasound^16^, high pressure microfluidization^17^ and gamma-irradiation^18^ have been used to prepare LMP. Chemical modification is most widely used approach for this purpose. Actually, the commercially available PYKTIN^®^ in the market was prepared by this approach^19^. However, chemical treatment is considered as a harsh process and can generate environmental pollution issues. Enzymic modification is a good alternative to generate LMP. However, the availability of specific enzymes to degrade pectin and the high cost limit its broad application in the industry^20^. Thermal treatment for LMP is clean and gentle, but its efficiency was low^12^.

Ultrasound has been recently reported a promising approach to degrade a diverse form of polymers including polysaccharides^16, 21^. As the preparation does not introduce secondary pollution, ultrasound approach has been considered as an effective and “green” technique^22^. Ultrasound breaks polymers by hydroxyl radicals (·OH), which are produced by the acoustic cavitation that involves the formation, collision and subsequent expansion of microbubbles during sonication^23^. The limitation of ultrasound is that, due to the attenuation of energy transmission under a prolonged or high-intensity ultrasonic field^24^, it can only produce polysaccharides fragments larger than 20 kDa. Therefore, attempts have been made to combine with other methods, such as H_2_O_2_^25^ and enzyme^22^ to try to increase the degradation efficiency of ultrasound. To our knowledge, however, neither H_2_O_2_^25^ nor enzyme^22^ can significantly promote the degradation efficiency of ultrasound.

In the waste-water industry, advanced oxidation process has been widely used, as an efficacious and clean technology to treat polluted water by degradation organic wastes such as azo dye^26^, phenolic compound^27^ and cellulose^28^ through the HO∙ generated during the reaction^29–31^. As one of the most promising advanced oxidation processes, Fenton reaction particularly utilizes ferrous ion as the catalyst to generate HO∙ from H_2_O_2_^30^ (as shown in the reaction (1)). Fenton process involves many complex mechanisms and several important parameters such as temperature and pH can influence this process^32^.

1 $${{\rm{Fe}}}^{2+}+{{\rm{H}}}_{2}{{\rm{O}}}_{2}\to {{\rm{Fe}}}^{3+}+{\rm{HO}}\cdot +{{\rm{OH}}}^{-}$$

Interestingly, Fenton process has been reported to combine with ultrasound to to improve the degradation efficiency during wastewater treatment^33, 34^. However, to our knowledge, there is no report in combining ultrasound with Fenton system to prepare functional polysaccharides with small molecular size.

In this study, we combined ultrasound with Fenton process to prepare ultra-low molecular pectin with much improved degradation efficiency and improved anti-oxidant activity. Multiple physical and chemical approaches such as monosaccharide composition analysis, degree of esterification, FT-IR and NMR spectroscopy were adopted to delineate the structure of degraded product and the mechanism of US-Fenton process to efficiently degrade pectin was proposed.

## Results and Discussion

### Fenton process efficiently generates low molecule pectin and the ultrasound treatment can further accelerate this process

**Figure 1:**
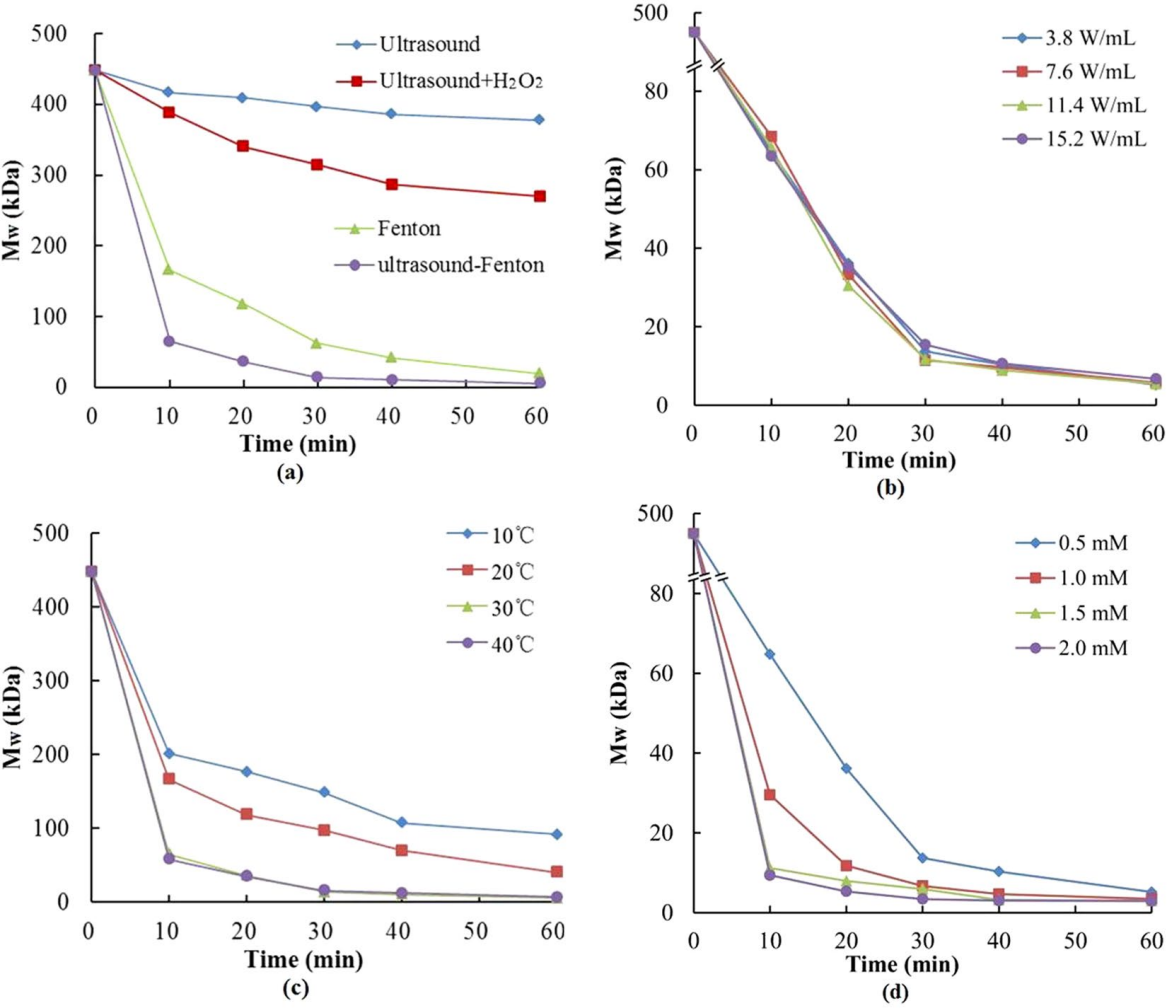
Comparision of citrus pectin degradation by (**a**) different degradation systems and US-Fenton system with different (**b**) ultrasonic intensities, (**c**) temperatures and (**d**) Fe^2+^ concentrations.

We first examined whether Fenton process can degrade pectin. As shown in Fig. 1a, single Fenton (0.5 mM Fe^2+^ and 6 g/L H_2_O_2_ at 30 °C) treatment degraded citrus pectin from 448 kDa to 19.78 kDa in 60 minutes (Fig. 1a). In stark contrast, ultrasound (3.8 W/L), either alone or combined with H_2_O_2_ (6 g/L), exhibited limited degradation effect. Even when combined with H_2_O_2_ (6 g/L), ultrasound failed to degrade pectin into fragments below 250 kDa within the same time frame. This result clearly demonstrates that Fenton process is a much more efficient approach to produce LMP when compared to ultrasound-based methods.

Next, we determined whether ultrasound can further enhance Fenton process to degrade pectin. Surprisingly, The combination of ultrasound and Fenton (US-Fenton) not only significantly accelerated the degradation process but also greatly improved the degradation efficiency, as demonstrated by the appearance of much smaller 5.2 kDa products within 60 minutes. This 5.2 kDa LMP has never been largely detected when either single approach was applied, indicating that US-Fenton process can synergize with each other to degrade pectin with much improved efficiency. This synergistic effect is likely due to the addictive effect of ultrasonic treatment and ferrous irons to generate more **·**OH radicals^35^. Taken together, our results clearly demonstrate that the Fenton process can efficiently produce LMP and ultrasound treatment can significantly improve this process.

### Optimization of the US-Fenton approach for pectin degradation

To further explore the optimal parameters to produce LMP, we next examined the effect of ultrasonic power, reaction temperature and the concentration of ferrous irons on the degradation efficiency.

Firstly, we examined the effect of ultrasonic power as it has been considered as an important operational parameter in sonochemical processes by controlling the formation of ·OH radicals and cavitation bubbles^36^. Interestingly, ultrasonic intensity exhibited no detectable effects on pectin degradation within the range of 3.8–15.2 W/mL (Fig. 1b), suggesting that ultrasonic power is not a critical parameter to for the efficient degradation of pectin in our system. Our results also implicate that the ultrasound accelerates Fenton process to degrade pectin mainly by functioning as a catalyst for the generation of free radicals, and low energy is sufficient to achieve this purpose. To save energy, the ultrasonic power of 3.8 W/mL was selected for the text of other parameters described below.

Next, we examined the effect of temperature on the US-Fenton reaction. As shown in Fig. 1c, degradation efficiency increases when the reaction temperature was elevated from 10 to 30 °C, consistent with the assumption that higher temperature can form more cavitation bubbles to degrade the organics during the ultrasonic treatment^37^. Nevertheless, no further improvement in the degradation efficiency was observed when the temperature was elevated to 40 °C. This observation may likely due to the sedimentation of Fe^2+^ and self-decomposition of H_2_O_2_^38^ under high temperature, which reduce in the degradation efficiencies.

Finally, we investigated whether the concentration of ferrous irons can affect the degradation efficiency. As shown in Fig. 1d, the degradation efficiency was significantly improved when Fe^2+^ concentration increased from 0.5 mM to 2.0 mM, suggesting that more Fe^2+^ can benefit the formation of ·OH radicals. The same observation was also observed by others^29^. However, there was a slight difference in molecular weight among 1, 1.5 and 2 mM in the 40–60 mins and the degradation efficiency slowed down because of the consumption of H_2_O_2_. So 1 mM Fe^2+^ was considered to be a suitable concentration.

In conclusion, US-Fenton process could cause rapid degradation of citrus pectin, which attributes to the fact that the generated **·**OH radicals could react with pectin by abstracting a hydrogen atom, resulting in the depolymerization of sugar chain. The ultrasonic temperature and ferrous concentration greatly affect on the degradation efficiency, while only very low intensity power is needed for accelerating the Fenton process and degradation of the pectin.

### US-Fenton process produces LMPs with relatively high homogeneity

**Figure 2:**
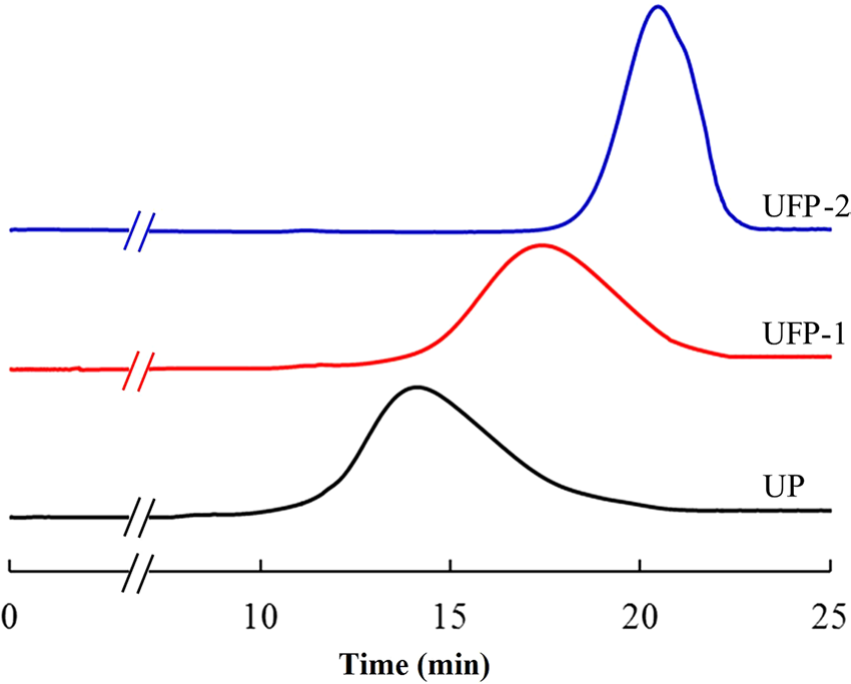
HPSEC chromatography of citrus pectin and its degraded pectins by US-Fenton treatment from 0 to 25 min on a ultrahydrogel TM 500 Column.

Notably, by adjusting the duration of treatment, the optimized US-Fenton process can generate two forms of degraded pectin with distinct molecular weights (Fig. 2). Pectin was degraded from 448.26 kDa to 53.52 kDa by US-Fenton within 5 minute**s**. When the process was prolonged to 35 minutes, pectin were cleaved into 5.5 kDa product. The 53.52 kDa and 5.5 kDa products were defined as “US-Fenton-degraded Pectin (UFP)-1 and UFP-2”, respectively.

To further determine the homogeneity of UFP-1 and UFP-2 products, we calculated the polydispersion index (PI) of UP, UFP-1 and UFP-2 by performed HPSEC chromatography. PI is a frequently used indicator for overall sharpness or breadth of polysaccharide in its molecular weight range. As shown in Fig. 2, the PI values of UP, UFP-1 and UFP-2 were 3.57, 2.98 and 1.59, respectively, indicating that US-Fenton treatment affected pectin’s structural molecular distribution. More importantly, HPSEC reveals that the molecular weight distribution of the degraded products, in particular UFP-2 was much “sharper” compared to UP, which indicates our optimized US-Fenton process degrades pectin polysaccharide into low molecular weight fractions with relatively high homogeneity.

### Monosaccharide composition analysis reveals the enrichment of RG-I domain in the US-Fenton modified citrus pectin

Despite pectins derived from variant resources or prepared by different methods can exhibit diverse structural characteristics, all of them can be considered as the combination of three types of domains or “building blocks”: homogalacturonan (HG), rhamnogalacturonan-I (RG-I) and rhamnogalacturonan-II (RG-II). These domains can be distinguished based on their monosaccharide composition^39^.

We aimed to determine whether the degraded pectin products harbor HG, RG-I and RG-II domains with distinct abundance. As expected, galacturonic acid (GalA), which constitutes as the backbone of all three domains, was shown as the major monosaccharide in both UP and its degraded products UFP-1 and UFP-2 (see Table 1). In addition, all other monosaccharides reported in pectin, such as galactose (Gal), rhamnose (Rha), arabinose (Ara), glucose (Glc), Fucose (Fuc) and mannose (Man) were detected in all three forms of pectin, implicating that US-Fenton treatment did not alter the panel of monosaccharide types.

**Table 1:** Monosaccharide composition of different pectin polysaccharide.

Monosaccharides	UP	UFP-1	UFP-2
Man	0.45 ± 0.02	1.52 ± 0.12	1.85 ± 0.04
Rha	8.22 ± 1.13	20.97 ± 1.96	23.36 ± 0.82
GalA	65.42 ± 3.29	35.63 ± 1.45	29.38 ± 2.26
Glc	4.42 ± 0.41	9.97 ± 0.05	11.15 ± 0.43
Gal	15.2 ± 0.01	22.39 ± 0.96	24.21 ± 2.47
Ara	4.66 ± 0.14	7.67 ± 0.00	8.14 ± 0.68
Fuc	1.63 ± 0.08	1.85 ± 0.21	1.91 ± 0.01
Rha/GalA	0.13	0.59	0.80
(Gal + Ara)/Rha	2.42	1.43	1.38
GalA/(Fuc + Rha + GlcA + Ara + Gla + Xyl)	2.20	0.67	0.51

Each value represents the mean of three replicates. Rha/GalA: the contribution of RG to pectin population; (Gal + Ara)/Rha: the length of side chains attached to RG-I; GalA/(Fuc + Rha + GlcA + Ara + Gla + Xyl): the linearity of pectin.

Despite existing in all samples, different monosaccharide types exhibited quite distinct relative abundance. In particular, the total mol% of neutral sugars such as Rha, Glc, Gal and Ara markedly increased after US-Fenton treatment. Meanwhile, the mol% of GalA decreased accordingly. As GalA is much more enriched in the HG domain, these data suggest that the HG domain is more liable to be attacked by US-Fenton induced **·**OH.

Furthermore, the Rha/GalA ratio increased but (Ara + Gal)/Rha ratio decreased after pectin was degraded. As the molar ratio of Rha/GalA reveals the relative abundance of the main chain of pectin and that of (Ara + Gal)/Rha reflects the average size of the neutral side chains^40^, such alterations suggest the breakage of the backbone HG regions and the cleavage of the RG-I region. Compared to the HG domain, the RG-I region were only slightly affected, further supporting the notion that indicating free radicals generated by US-Fenton preferentially attack at the HG region of pectin.

Finally, the mole ratio of the GalA/(Fuc + Rha + GlcA + Ara + Gla + Xyl) significantly decreased after treatment. As this ratio reflects the linearity of pectin, this observation clearly demonstrates that UPF-1 and UFP-2 products showed much less of linearity compared to the unfragmented pectin. Taken together, our analyses based on the monosaccharide composition strongly support the notion that the US-Fenton treatment preferentially degrades the HG region, therefore enriches the RG-I domain after the unfragmented pectin was converted to the smaller UPF-1 and UFP-2 products.

### US-Fenton induced degradation decreases the Degree of esterification of pectin

The molecular weight, content of GalA and degree of esterification (DE) are three fundamental characteristics of pectin chemistry, which have great impacts on the gelling, stabilizing and thickening property of pectin. Thus far we have examined the molecular weight and content of GalA. Here we determined the alteration of DE after US-Fenton treatment (Table 2). DE is composed of the degree of methoxyl (DM) and degree of acetylation (DA). Our data revealed that, after degradation, methoxyl level was significantly decreased. This observation indicates that methyl groups from pectin chain were removed the reaction. It is worth of mentioning that, as the DM was less than 50%, both in UFP-1 and in the UFP-2 productscan be classified as low-methoxyl (HM) pectin based on the definition^41^.

**Table 2:** The content of galacturonic acid (GalA%), degree of methylation (DM), actylation (DA) and the Mw of different pectin polysaccharides.

Sample	Mn (kDa)	Mw (kDa)	PI (Mw/Mn)	GalA%	DM (%)	DA (%)
UP	125.48	448.26	3.57	82.7	55.32	5.63
UFP-1	25.75	53.52	2.98	53.12	36.76	4.12
UFP-2	3.46	5.50	1.59	44.41	30.35	3.77

PI: polydispersity index.

### FT-IR spectroscopy analysis validates the decrease of pectin DM after the US-Fenton process

FT-IR spectroscopy analysis was further performed to validate the alteration of the degree of methylation (DM) after US-Fenton process^42^. In the FT-IR spectra of pectin (Fig. S1), the two major absorption at around 3425 cm^−1^ and 2938 cm^−1^ were caused by stretching of hydroxyl groups and C–H stretching of CH_2_ groups, respectively. And two featured absorptions at 1762 cm^−1^ and 1652 cm^−1^ were clearly observed, corresponding to the C=O stretching vibration of methylesterified carboxyl groups (COO-R) and the C=O stretching vibration of ionic carboxyl groups (COO-) respectively. The degree of methylation (DM) can be determined based on the ratio of the peak area at 1762 cm^−1^ over the sum of the peak areas of 1762 cm^−1^ and 1652 cm^−140^. Consistent with monosaccharide composition analysis described above, Fig. S1 clearly shows that the DM of citrus pectin drastically decreased after US-Fenton processing. Of note, although the content of β-D-mannose did not change (based on the featured absorption at 784 cm^−1^), FT-IR spectroscopy indicates that the composition of D-glucopyranosyl decreased significantly (based on the featured absorption at 873 cm^−1^) after US-Fenton process.

### NMR Spectra

**Figure 3:**
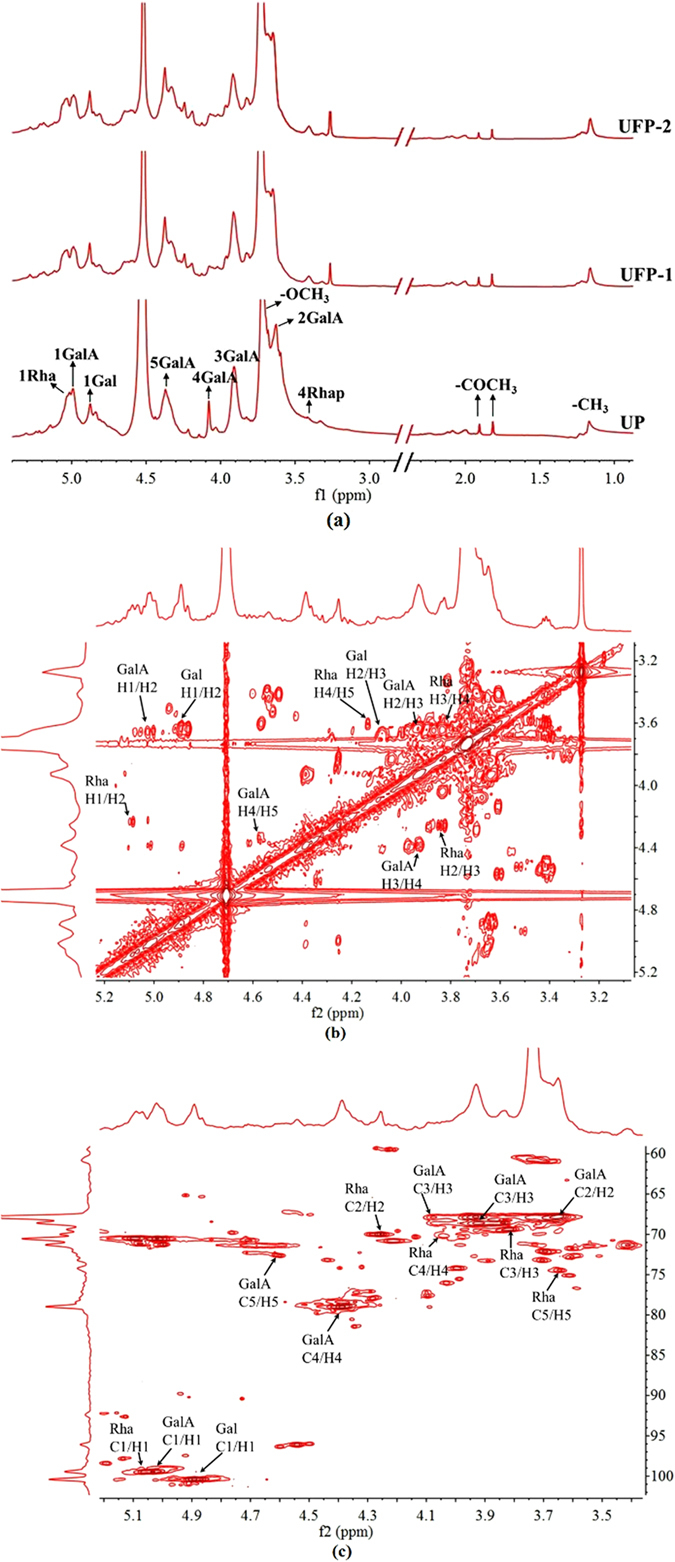
NMR spectra of pectin. (**a**) ^1^H NMR spectra of UP, UFP-1 and UFP-2 and (**b**) COSY and (**c**) HSQC spectra of UPF-2.

To better understand the structural information of UP, UFP-1 and UFP-2 products, we performed the ^1^H NMR (Fig. 3a). A couple of proton signals can be unambiguously assigned in the ^1^H NMR spectra of the samples. For instance, in the ^1^H NMR spectra of natural pectin, the highest peak at 3.73 ppm was derived from methyl groups binding to carboxyl groups of GalA. Two signals around 1.90 ppm were derived from acetyl groups binding at 2-O- and 3-O-GalA. The former was at 1.91 ppm and the latter was at 1.82 ppm. Signals at 1.22 ppm and 1.16 ppm were instead derived from methyl groups of L-rhamnose and were assigned to the O-2 and O-2, 4 linked rhamnose, respectively. Other major signals in nature pectin were assigned to the five proton in the D-galacturonic acid (H-1, 4.99 ppm; H-2, 3.63 ppm; H-3, 3.91 ppm; H-4, 4.08 ppm and H-5, 4.37 ppm). In the anomeric region, the signals at 5.03 ppm and 4.88 ppm were attributed to the H-1 of rhamnose and H-1 of galactose, respectively.

Compared to that of the intact pectin, the ^1^H NMR spectra of US-Fenton-treated pectin UFP-1 and UFP-2 exhibited an evident decrease in the intensity of the anomeric peak at 4.99 ppm and the increases of two anomeric signals at 5.03 ppm and 4.88 ppm. These alterations were much more profound in the UFP-1 and UFP-2 products and may be attributed to the selective cleavage of galacturonic acid catalyzed by the US-Fenton reaction. Therefore, our results from the ^1^H NMR analysis suggests that **·**OH produced by US-Fenton process preferentially attacks the glycosidic bond without altering the overall pectin structural features.

In view of the low resolution of the ^1^H NMR spectra, we next employed 2D NMR (COSY (Fig. 3b), HSQC (Fig. 3c) and TOCSY (Fig. S2)) to further determine the chemical structure of the degraded fraction UFP-2 by assigning the chemicals shifts. As shown in Fig. 3b and c, the COSY, TOCSY and HSQC spectra of UFP-2 revealed three distinct correlation signals in the anomeric region at 5.09 ppm/99.46 ppm, 5.02 ppm/99.46 ppm and 4.89 ppm/100.37 ppm, which can be assigned to H-1 of GalA, Rha and Gal respectively based on the previous studies^43, 44^. All chemical-shifts assignments were shown in Table 3.

**Table 3:** 1H and ^13^C chemical shifts in the 1D and 2D NMR spectra of UFP-2.

Glycosyl Residue	H1	H2	H3	H4	H5	H6
	(C1)	(C2)	(C3)	(C4)	(C5)	(C6)
GalA	4.99	4.63	3.93	4.37	4.52	nd
	99.46	67.82	68.61	78.99	72.79	nd
Rha	5.09	4.26	3.83	3.64	4.04	nd
	99.46	70.09	69.35	74.45	70.24	nd
Gal	4.89	3.64	4.08	3.91	4.38	3.63
	100.47	nd^a^	67.96	nd	78.33	67.82

^a^Nd-not detected.

### The proposed mechanism of degradation of citrus pectin

**Figure 4:**
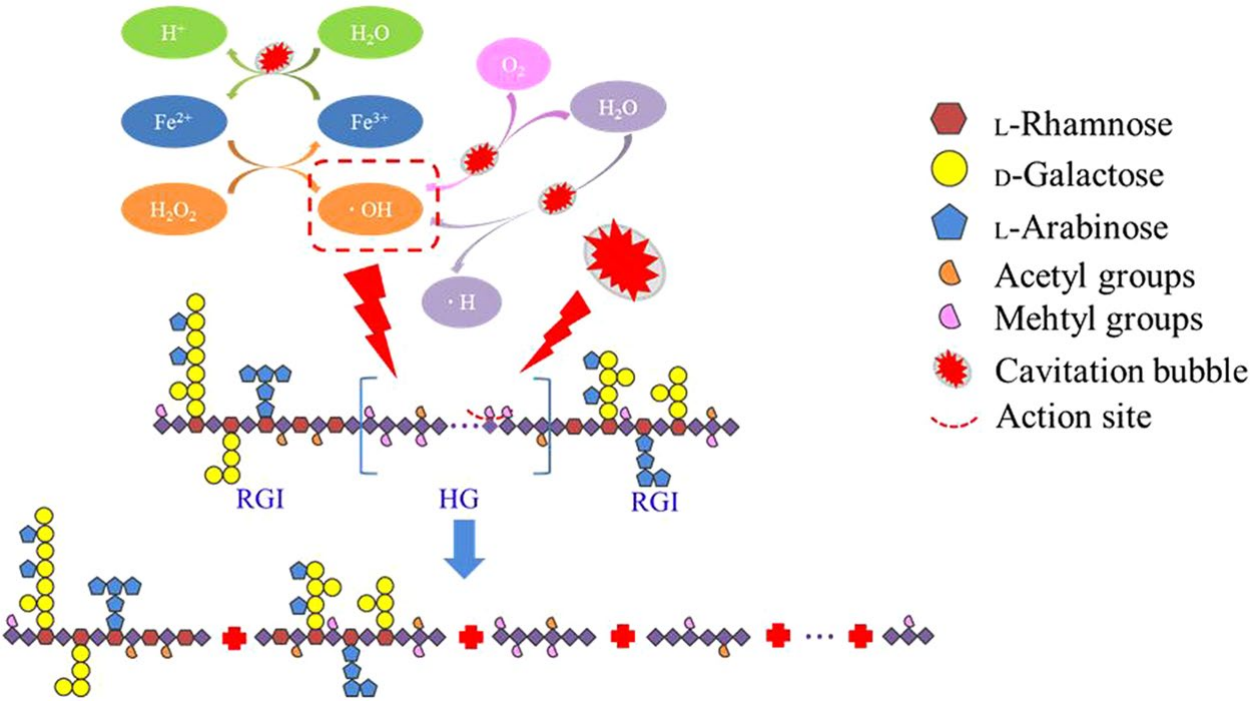
The selective shear schematic diagram of pectin degradation under ultrasound accelerated Fenton process.

Based on the detailed analysis of chemical composition, IR and NMR, we propose a tentative model to explain the mechanism of US-Fenton process to generate RG-I enrich fragments and the synergistic effect of ultrasound and the Fenton reaction to achieve a high efficiency in the degradation of pectin. As shown in Fig. 4, ·OH free radicals are generated during the reaction. We reasoned that the reaction preferentially attacks the HG domain, therefore the RG-I domain was largely enriched after degradation. Ultrasound cleaves the C-O bond of ester linkages and the **·**OH produced by US-Fenton reacts with thee methyl ester bonds to break its C-O bond^16, 21^, both of which lead to the decrease of DE of pectin.

The ultrasound accelerates the decomposition of H_2_O_2_, therefore promotes the catalytic efficiency of Fe^2+^ in the reaction solution. On the other hand, decomposition of H_2_O_2_ provided abundant oxygen which further boost the formation of cavitation bubbles. And ultrasonic process can enhance the conversion of Fe^3+^ into Fe^2+^ in reaction solution, promoting Fenton system to produce more **·**OH to act on the polysaccharide chains^35^.

### Antioxidant activities

**Figure 5:**
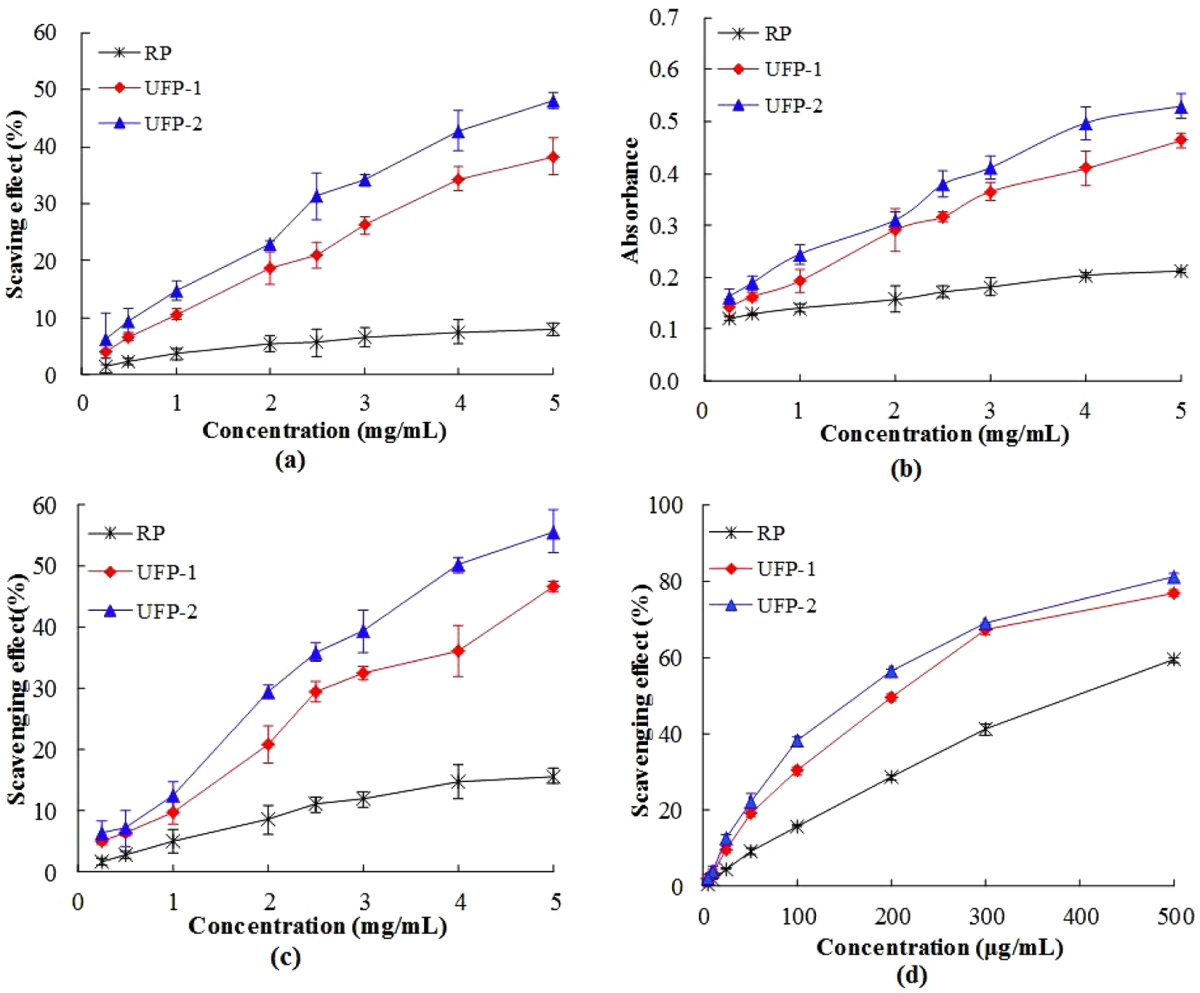
Antioxidant activities of pectin and US-Fenton degraded fractions (UFP-1 and UFP-2). (**a**) DPPH radical scavenging activity, (**b**) FRAP assay, (**c**) ABTS assay and (**d**) ORAC assay.

In view that the antioxidant activity is one of the most important indexes of pectin and its degraded products bioactivities, we next determined whether the US-Fenton treatment affects the antioxidant activity of natural pectin. Surprisingly, in all different types of antioxidant assays (DPPH radical scavenging activity (Fig. 5a), FRAP assay (Fig. 5b), ABTS assay (Fig. 5c) and ORAC assay (Fig. 5d)), the US-Fenton degraded products, in particular UFP-2 exhibited much increased antioxidant activity compared to the original pectin. Such an increase in the antioxidant activity can be explained by the better water-solubility and larger surface area of contact after the pectin molecule was degraded to smaller fragments, which in turn have more chance to contact with free radicals^45^. Further studies are warranted to investigate the antioxidant effects of US-Fenton generated LMP such as UFP-2 by using both cell culture and *in vivo* animal models.

### Concluding remarks

In this study, we reported an ultrafast and convenient method to prepare RG-I enriched ultra-low molecular weight pectin. In our approach, we combined both ultrasound and Fenton reaction. We found that the combination of both approaches was much more effective than any single approach was applied. We also determined the optimal conditions to produce LMP by mapping out the best parameters for ultrasonic intensity, the concentration of ferrous ion and the temperature. Chemical composition, FT-IR and NMR analysis were applied to analyze the composition and structural features of US-Fenton degraded products. Our results suggest that the HG domain was more likely to be attacked via hydroxyl radical but the RG-I domain was enriched, which may contribute to the elevated antioxidant activity of degraded pectin produced by our approach. In summary, we described the US-Fenton reaction as a green and efficient method for the preparation of RG-I enriched ultra-low molecular weight pectin. Further studies should focus on the biological activity of RG-I enriched ultra-low molecular weight pectin at the cellular and whole body level.

## Materials and Methods

### Material and chemical reagents

Citrus pectin and dextrans were purchased from Sigma-Aldrich (St. Louis, MO, USA). Hydrogen peroxide, ferrous chloride tetrahydrate, HPLC-grade methyl alcohol and deuterium oxide were obtained from Sinopharm Chemical Reagent Co., Ltd. (Shanghai, China). 2,2-diphenyl-1-picrylhydrazyl (DPPH), 2,20-Azobis(2-methylpropionamidine) dihydrochloride (AAPH), 6-hydroxy-2,5,7,8-tetramethyl-chroman-2-carboxylic acid (Trolox) and 2,2′- azino-bis-(3-ethylbenzo-thiazoline-6-sulfonic acid) diammonium salt (ABTS) were acquired from Aladdin Chemical Reagent Co., Ltd. (Shanghai, China).

### Ultrasound accelerated Fenton processing of citrus pectin

#### Comparison of US-Fenton with other treatments

Ultrasound treatments were performed (Scientz-IID, Ningbo Scientz Biotechnology Co., Ningbo, China) with the following parameters: maximum ultrasound power output, 900 W, frequency, 22 kHz, intermittent type, 2 s on and 2 s off, and horn micro tip diameter, 10 mm. Twenty-five milliliters of pectin solution (5 mg/mL) were put in a cylindrical glass reactor (Φ, 2.90 cm) and the generator probe was submerged (about 1 cm below the liquid surface) to release ultrasonic energy.

Under the certain condition, US-Fenton (ultrasonic intensity, 3.8 W/mL, the concentration of ferrous, 0.5 mM and the dosage of H_2_O_2_, 6 g/L) was compared with single ultrasound treatments (3.8 W/mL), ultrasound (ultrasonic intensity, 3.8 W/mL) assisted with H_2_O_2_ (6 g/L), and single Fenton system (the concentration of ferrous, 1 mM and the dosage of H_2_O_2_, 6 g/L). All the tests were performed at the temperature of 30 °C for 60 min.

#### Optimization of US-Fenton factors

The effects of the following parameters were investigate: ultrasound intensity (3.8, 7.6, 11.4 and 15.2 W/mL), temperature (10, 20, 30 and 40 °C) and ferrous concentration (0.5, 1.0, 1.5 and 2 mM). The general degradative condition of all treatments were as follow: reaction time of 60 min, temperature at 30 °C, Fe^2+^ concentration of 0.5 mM, hydrogen peroxide of 6 g/L, the ultrasound intensity of 3.8 W/mL.

### Determination of the Average Mw and Molecular Weight Distribution

The Mw and polydispersity index (PI) of pectin samples were determined by a high performance size exclusion chromatography (HPSEC) system according to the method described by Guo with some modifications^46^. The average Mw determination was performed on a Waters 1525 HPLC system (Waters, Milford, USA) with ultrahydrogel TM 500 Column (Waters, Milford, USA). Forty microliters of the pectin solutions (2.0 mg/mL) were injected and eluted by 0.2 M NaCl at a flow rate of 0.5 mL/min. The standard dextrans of 1.27, 11.60, 5.22, 48.60, 147.60, 273.00, 409.80 and 667.80 kDa were used to obtain the calibration curves. The average Mw and PI of pectin samples and standard dextrans was calculated using the Breeze 2 software.

### Determination of Monosaccharide Composition

The monosaccharide content of citrus pectin was determined using PMP-HPLC method described by Wang^47^. In brief, pectin sample (3 mg) was hydrolyzed with 2 M trifluoroacetic acid at 110 °C for 8 h. The hydrolyzed samples (400 μL) were dissolved into 0.3 M NaOH (450 μL) and 0.5 M methanol solution of PMP (450 μL). Lactose (50 μL) was added as an internal standard before derivatization. The mixture was allowed to react for 30 min at 70 °C before cooling to room temperature. Then the resulting solution was neutralized with 450 μL HCl (0.3 M) and extracted by 1 mL of chloroform twice. Then the aqueous layer was filtered through a 0.45 μm membrane before HPLC analysis.

Analysis of the PMP-labeled monosaccharides was carried out by a Waters 2695 HPLC system (Waters, US) equipped with a PDA 2996 detector (Waters, US). A Zorbax Aclips XDB-C18 column (4.6 mm × 250 mm, 5 μm, Agilent, USA) was used. The mobile phase was 0.05 M sodium phosphate (pH 6.9) with (A) 15% and (B) 40% acetonitrile, using a linear gradient elution of 0–15% (0–10 min) then 15–25% buffer B (10–30 min).

### The determination of degree of esterification

Degree of methoxylation (DM) and degree of acetylation (DA) were determined using the reported method with some modifications^48^. Pectin samples (10 mg) were saponified at 4 °C for 1 h. Reaction mixtures were centrifuged and supernatant was adjusted pH to 2 by adding to HCl. The mixed standard solution consisted of 100 μL/mL isopropanol (10 μL), 100 μL/mL acetic acid (10 μL), methanol (6 μL) and ultrapure water (2 mL). Methoxyl and acetyl groups were quantified by a HPLC system equipped with a C18 column (4.6 mm × 250 mm, 5 μm, Elite, China).

Besides, the standard solutions of galacturonic acid (0, 10, 20, 40, 60, 80, 100 and 120 μg/mL) were prepared and measured by sulfate-carbazole method at 734 nm. The result were expressed through standard curve, y = 0.0032x+ 0.0214, R^2^ = 0.999, where y represents the absorbance value, x represents galacturonic acid concentration (μg/mL). The galacturonic acid of pectin samples was determined by the same method and their contents were calculated according to standard curve. The value of galacturonic acid was used to calculate degree of esterification. DM and DA were calculated by the following equations:

2 $${\rm{FR}}=\frac{{{\rm{M}}}_{{\rm{MeOH}}{\rm{or}}{\rm{AcOH}}}}{{{\rm{M}}}_{{\rm{IPA}}}}\times \frac{{{\rm{A}}}_{{\rm{IPA}}}}{{{\rm{A}}}_{{\rm{MeOH}}{\rm{or}}{\rm{AcOH}}}}$$

3 $${\rm{DM}}={\rm{FR}}\times \frac{{{\rm{A}}}_{{\rm{MeOH}}}{{\rm{M}}}_{{\rm{IPA}}}}{{{\rm{A}}}_{{\rm{IPA}}}{{\rm{M}}}_{{\rm{Sample}}}}\times \frac{176\times {10}^{4}}{\mathrm{GalA} \% \times 32}$$

4 $${\rm{DA}}={\rm{FR}}\times \frac{{{\rm{A}}}_{{\rm{AcOH}}}{{\rm{M}}}_{{\rm{IPA}}}}{{{\rm{A}}}_{{\rm{IPA}}}{{\rm{M}}}_{{\rm{Sample}}}}\times \frac{176\times {10}^{4}}{\mathrm{GalA} \% \times 60}$$

where FR is factor responses for acetic acid and methanol; M_MeOH or AcOH or IPA_ and A_MeOH or AcOH or IPA_ are the mass and peak area of MeOH, AcOH or IPA, respectively; GalA% is the content of galacturonic acid.

### FT-IR and NMR spectroscopy

The FT-IR spectra of citrus pectins was recorded on a Nicolet 5700 spectrometer (Thermo Fisher Scientific, USA) using the absorbance mode in a frequency range of 4000–400 cm^−1^, with a resolution of 4 cm^−1^. The dried pectins were mixed with KBr powder (50 mg) and pressed into KBr pellets prior to FT-IR analysis. The data was analysed using the Ominic 7.2 software.

For NMR analysis, citrus pectin and low molecular weight fractions (35 mg) were evaporated with 550 μL of D_2_O (99.96%) twice via vacuum freeze drying before final dissolution in 500 μL of high-quality D_2_O (99.96%) containing 0.1 μL of acetone. The ^1^H NMR spectra was collected by a Bruker AVIII 800 M (Switzerland) at room temperature. Two-dimensional spectra COSY, TCOSY and HSQC were recorded using the standard Bruker procedures. The number of scans (ns) in each experiment was dependent on the sample concentration.

### Antioxidant activity assay

#### DPPH free-radical scavenging activity assay

The samples on scavenging DPPH free-radical was studied using the modified method described by Guo^49^. Briefly, twelve microliters of pectin solutions were incubated with 0.1 mM DPPH in anhydrous ethanol (188 μL) in a 96-well micro plate plates. The reaction mixture reacted at room temperature for 30 min. The absorbance of the sample solutions were determined at 517 nm. The DPPH activity was calculated by the following equation:

5 $${\rm{Scavenging}}\,{\rm{effect}}\,( \% )=(1-{{\rm{A}}}_{{\rm{sample}}517}{/{\rm{A}}}_{{\rm{control}}517})\times 100$$

#### Ferric-reducing antioxidant power (FRAP) assay

The reducing activities of samples were determined according to a previous method^50^ with some modifications. The ferric-tripyridyltriazine (FeIII-TPTZ) complex solution was prepared with 0.1 M acetate buffer (pH 3.6), 20 mM ferric chloride and 10 mM TPTZ (dissolved in 40 mM HCl) at a ratio of 10:1:1 (v/v/v). A volume of the FeIII-TPTZ solution (188 μL) was added to the pectin samples (12 μL) at 37 °C for 10 min. The absorbance was measured at 593 nm.

#### ABTS assay

The ABTS assay was measured according to the previous described method^51^ with some slight modifications. The ABTS^+^ cation solution consisted of 7 mM ABTS stock solution (25 ml) and 140 mM potassium persulfate solution (440 μL), which was reacted in the dark at room temperature for 14 h before using it. The diluted ABTS^+^ solution (an absorbance value of 0.7 ± 0.02 at 734 nm, 188 μL) was thoroughly mixed with pectin solutions (12 μL). The mixture was kept in the dark for 10 min and the absorbance was measured at 734 nm using Microplate Reader.

6 $${\rm{Total}}\,{\rm{antioxidant}}\,{\rm{capacity}}\,( \% )=(1-{{\rm{A}}}_{{\rm{sample}}734}{/{\rm{A}}}_{{\rm{control}}{\rm{734}}})\times 100 \% $$

#### Oxygen Radical Absorbance Capacity (ORAC) assay

The ORAC assay was executed according to a previous report^52^ with some modifications. Before the assay, a fluorescein solution (FLs) was prepared with 75 mM PBS at pH 7.4 (final concentration: 504 nM). Pectin solutions were dissolved in PBS. Subsequently, 25 μL of sample/Trolox solution and 25 μL FLs were mixed for 5 min at 37 °C, followed by the addition of 12.8 mM AAPH PBS solution (150 μL). The absorbance was measured for 3 h with the emission wavelength at 538 nm and excitation wavelength at 485 nm. The fluorescence decay curve (AUC) was integrated by Ascent Microsoft. The scavenging effect was calculated by the following equation.

7 $${\rm{Scavenging}}\,{\rm{effect}}\,( \% )=({{\rm{AUC}}}_{{\rm{control}}}-{{\rm{AUC}}}_{{\rm{AAPH}}}){/(\mathrm{AUC}}_{{\rm{sample}}}-{{\rm{AUC}}}_{{\rm{AAPH}}})\times 100 \% $$

## Electronic supplementary material

Fast preparation of RG-I enriched ultra-low molecular weight pectin by an ultrasound accelerated Fenton process
