# Supplementary material for: Fast preparation of RG-I enriched ultra-low molecular weight pectin by an ultrasound accelerated Fenton process
Source: Sci Rep. 2017 Apr 3;7:541. doi: 10.1038/s41598-017-00572-3 (PMC5428719; doi:10.1038/s41598-017-00572-3)
Supplement: Supplementary file 1 — Fast preparation of RG-I enriched ultra-low molecular weight pectin by an ultrasound accelerated Fenton process [file 41598_2017_572_MOESM1_ESM.pdf]

Fast preparation of RG-I enriched ultra-low molecular weight pectin by an ultrasound accelerated Fenton process

Zijian Zhi, Jianle Chen, Shan Li, Wenjun Wang, Rui Huang, Donghong Liu, Tian Ding, Xingqian Ye & Shiguo Chen*

Zhejiang University, College of Biosystems Engineering and Food Science, Fuli Institute of Food Science, Zhejiang Key Laboratory for Agro-Food Processing, Zhejiang R & D Center for Food Technology and Equipment, Hangzhou 310058, China.

The corresponding authors:

Tel. / Fax: 86-571-88982151 (Shiguo Chen); 86-571-88982155 (Xingqian Ye)

E-mail: chenshiguo210@163.com (Shiguo Chen); psu@zju.edu.cn (Xingqian Ye)

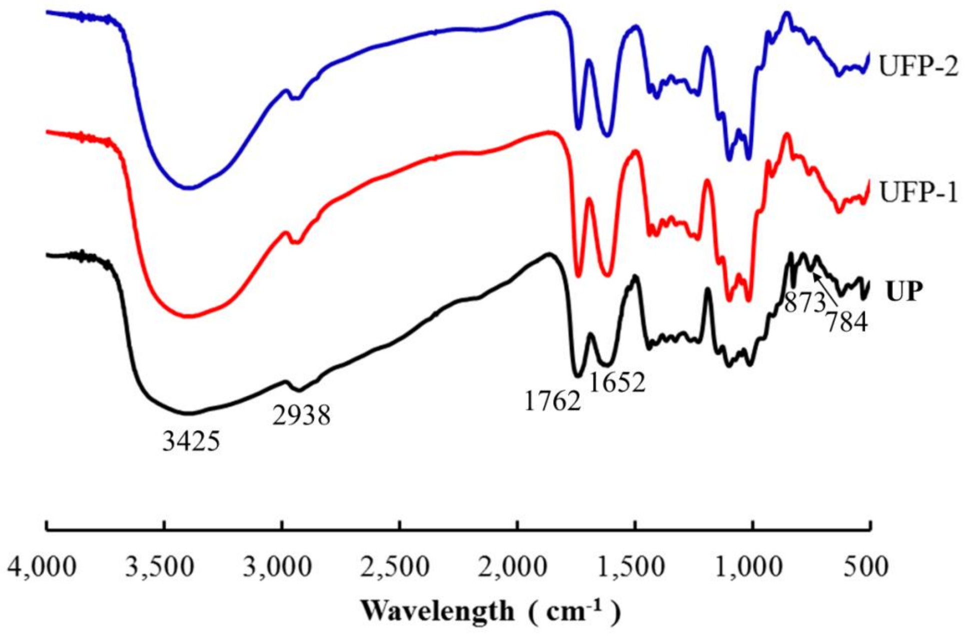

Fig.S1 FT -IR spectra of pectin polysaccharides.

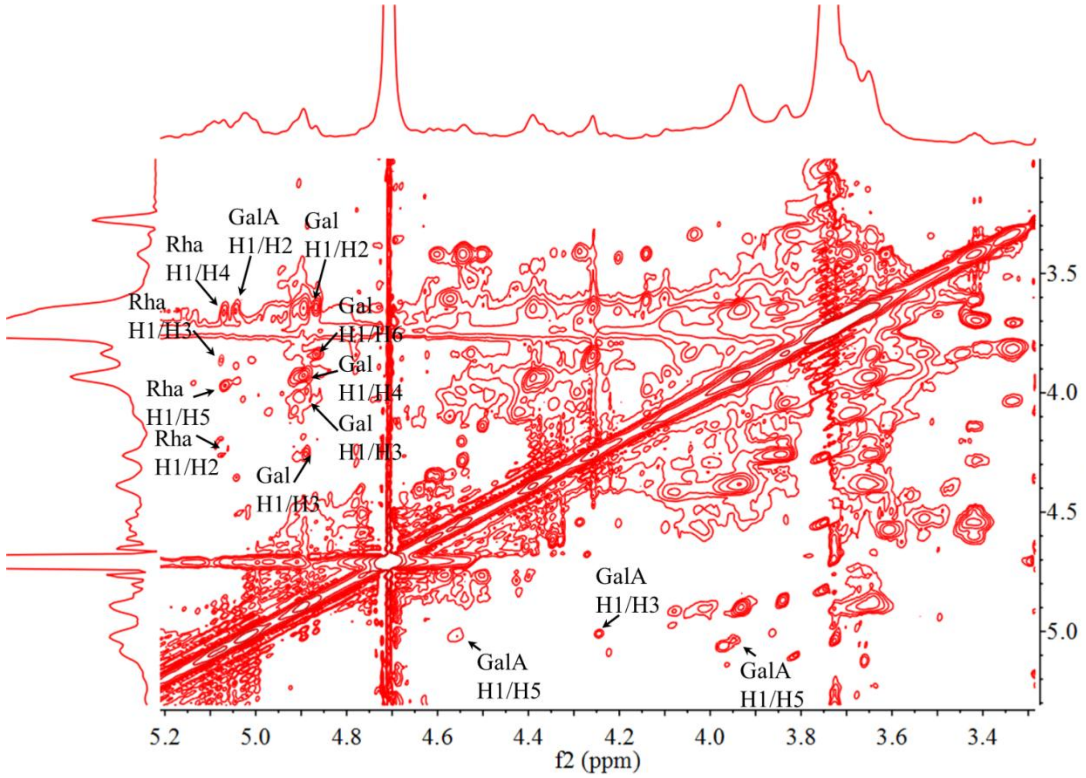

Fig.S2 TOCSY spectra of pectin polysaccharides.
